# ﻿Land of giants: two new pachycaul *Monstera* (Araceae, Monsteroideae, Monstereae) from Panama

**DOI:** 10.3897/phytokeys.267.170789

**Published:** 2025-12-04

**Authors:** Marco Cedeño-Fonseca, Orlando O. Ortiz, Alistair Hay, Alejandro Zuluaga

**Affiliations:** 1 Botanischer Garten und Botanisches Museum Berlin, Freie Universität Berlin, Königin-Luise-Straße 6-8, D-14195 Berlin, Germany Freie Universität Berlin Berlin Germany; 2 Centro de Investigación Jardín Botánico Lankester, Universidad de Costa Rica, P.O. Box 302-7050, Cartago, Costa Rica Universidad de Costa Rica Cartago Costa Rica; 3 Research Associate, Herbario Luis Fournier Origgi (USJ), Centro de Investigación en Biodiversidad y Ecología Tropical, Universidad de Costa Rica, Apdo.11501−2060, San José, Costa Rica Universidad de Costa Rica San José Costa Rica; 4 Universidad de Panamá, Herbario PMA, Estafeta Universitaria, Apdo. 3366, Panama City, Panama Universidad de Panamá Panama City Panama; 5 Coiba Scientific Station (COIBA AIP), Clayton, Ciudad del Saber, Panama City, Panama Coiba Scientific Station (COIBA AIP) Panama City Panama; 6 Royal Botanic Gardens Sydney, Mrs Macquarie’s Road, Sydney 2000, Australia Royal Botanic Gardens Sydney Sydney Australia; 7 Jardín Botánico de la Paz y Flora, Bitaco, Valle del Cauca, Colombia Jardín Botánico de la Paz y Flora Bitaco Colombia; 8 Departamento de Biología, Universidad del Valle, Calle 13 # 100-00, Cali, Colombia Universidad del Valle Cali Colombia

**Keywords:** Central America, cloud forests, Talamanca Mountain, taxonomy

## Abstract

The genus *Monstera* is a diverse group of nomadic vines endemic to the Neotropics, with its greatest diversity concentrated in the montane forests of southern Central America. The Cordillera de Talamanca, spanning southern Costa Rica and western Panama, has emerged as a hotspot for pachycaul *Monstera* species which exhibit large leaf and inflorescence sizes. This study presents the discovery and description of two new giant *Monstera* species from Panama: *M. corana* and *M. colossica*. Both species occur relatively close to *M. gigas* in the cloud forests of the southern Talamanca range, an area characterised by high humidity, persistent cloud cover and heterogeneous volcanic soils. We provide detailed morphological descriptions, field observations, distribution maps and comparisons with morphologically related taxa.

## ﻿Introduction

*Monstera* [Araceae Juss., Monsteroideae Schott, tribe Monstereae Engler (aka *Rhaphidophora* clade] (Cusimano et al. 2011; Zuluaga et al. 2019; Haigh et al. 2022) is a genus endemic to the Neotropics, representing a diverse group of nomadic vines, with its greatest diversity concentrated in the montane forests of southern Central America, nonetheless largely in Costa Rica and Panama (Madison 1977; Zuluaga et al. 2019; Cedeño-Fonseca et al. 2022). *Monstera* exhibits particularly high species diversity in the Cordillera de Talamanca, especially in southern Costa Rica and western Panama (Madison 1977; Cedeño-Fonseca et al. 2022). Recent discoveries of exceptionally large pachycaul *Monstera* species have drawn increasing scientific attention to this region, such as *M. alfaroi* Croat & M.Cedeño, *M. gigas* Croat, Zuluaga, M.Cedeño & O.Ortiz and *M. titanum* Croat, M.Cedeño & O.Ortiz, the last two taxa currently holding the genus records for the largest leaves and inflorescences, respectively (Cedeño-Fonseca et al. 2021). Other notable examples of very large species within the genus include *M. lechleriana* Schott from South America and *M. deliciosa* Liebm. from Mesoamerica.

Despite the recent discovery of several giant-leaved species, the full extent of species-level diversity within these montane forests remains underexplored, specifically amongst large-leaved, morphologically similar taxa, which often remain overlooked due to their challenging taxonomy and collection and remote habitats. The phenomenon of very large size in *Monstera* has yet to be formally studied. However, research on plant gigantism more broadly suggests that extreme organ size, particularly in leaves and stems, is often shaped by ecological, physiological and evolutionary pressures (Givnish 1988; Zotz et al. 2002; Blackman et al. 2005; Poberezhnaya and Kopanina 2009; Bernal et al. 2017; Cox and Burns 2017). In Panama, which harbours the majority of large *Monstera* species, these taxa are typically found in montane cloud forests (except *M. corana*, described here), particularly in regions with volcanic soils. *M. titanum*, for example, is confined to the outskirts of the inactive volcano El Valle de Antón in central Panama. Notably, in a single locality (Fortuna in western Panama), two very large species (*M. gigas* and the here-described *M. colossica*) have been documented growing in proximity. Recent studies have identified this region as one of the cloudiest areas in Central America and as a zone of high soil heterogeneity, both of which significantly influence the composition and distribution of plant communities (Dalling et al. 2021). The specific soil type found in these sites, classified as Mafic-Volcanic, is characterised by its relatively high bulk density and clay content as well intermediate fertility (Turner and Dalling 2021). Soil traits, combined with consistently high humidity and/or cloud cover, may represent key environmental factors contributing to the evolution of very large species in *Monstera*. However, the evolutionary and functional significance of these traits remains poorly understood, highlighting the need for integrative studies that explore the morphological, ecological and genetic underpinnings of the very large size in this genus.

Through an approach combining extensive fieldwork and detailed herbarium-based morphological analysis, we identified two previously undescribed pachycaul species of *Monstera* from Panama. These discoveries contribute to the growing recognition of the Talamanca Mountain Range as a centre of diversity and endemism for Araceae, particularly within the subfamily Monsteroideae.

## ﻿Material and methods

Morphological comparisons were based on a careful examination of both herbarium specimens and living plants in the field. Further, all relevant taxonomic literature from Central and South America, type specimens from JSTOR Global Plants (2022) and protologues were examined. To compare morphological variation and to delimit the new species from closely-similar species, the following Herbaria were consulted (acronyms follow Thiers (2025) [continuously updated]): AGUAT, B, BM, BIGU, CHIP, COAH, COL, CR, CSAT, CUVC, DUKE, ENCB, F, HEM, HLDG, HNMN, HUAZ, ITIC, JAUM, JBB, JVR, K, LAGU, LSCR, MA, MEXU, MHES, MO, NY, PMA, SEL, SCZ, TEFH, UDBC, UCH, UJUAT, USCG, USJ, XAL, WU, as well as images of specimens accessible on-line at C, COL, EAP, ENCB, MEXU and TEFH.

Live plants were studied and photographed *in situ* with a digital camera Nikon COOLPIX P530 Sony A6000, as well as mobile phones with integrated high-resolution cameras, such as Huawei p20, iPhone 11 Pro and Samsung Galaxy S22 Ultra. Measurements in the description consist of a mix between living plants and herbarium specimens. All images and plates were processed in Adobe Photoshop v. 24.0.1 (Adobe Inc., San Jose, USA). The specimens from different localities were pressed and deposited at PMA.

Due to the high demand for aroid species such as ornamental plants and a rapidly growing black market that endangers native populations (even in protected areas), geographic coordinates are here omitted from all specimen citations and no distribution maps are provided.

### ﻿Taxonomic treatment

#### ﻿ Monstera corana

Taxon classificationPlantaeAlismatalesAraceae

M.Cedeño & O.Ortiz sp. nov.

A53952DB-436C-5927-B21B-D98363129724

urn:lsid:ipni.org:names:77372764-1

Figs 1, 2, 3

##### Type.

Panama • Provincia Bocas del Toro: distrito Chiriquí Grande, corregimiento Chiriquí Grande, áreas abiertas alrededor de la carretera principal, 17 November 2022, 85 m elev., *M. Cedeño, O. Ortiz, N. Köster, A. Hay & R. da pena 2730* (holotype PMA!; isotype B!).

**Figure 1.**
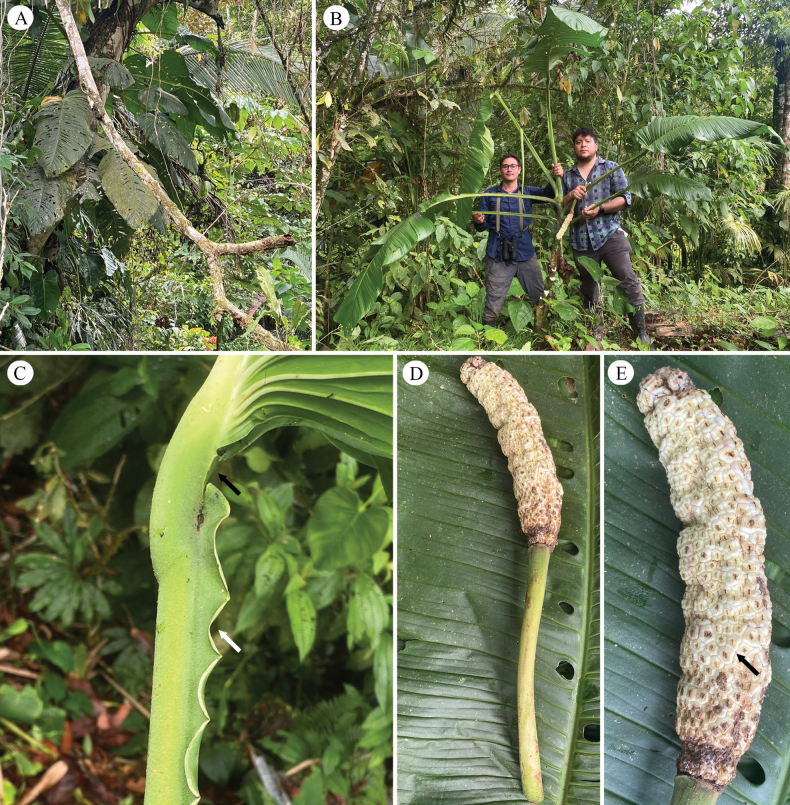
*Monstera corana* M. Cedeño & O. Ortiz. A. Adult plant growing in the forest around Chiriquí Grande; B. The first and second authors with the adult plant in the type locality – Photo by A. Hay; C. Petiole sheath thin and undulate (white arrow) and the geniculum flattened adaxially and transversally convex abaxially (black arrow); D. Infructescence in development; E. Spadix with the white stylar caps after anthesis and the styles truncate (black arrow). Photos by M. Cedeño.

##### Diagnosis.

*Monstera corana* differs from *M. buseyi* Croat & Grayum by having white, connected and consistently branched primary lateral veins (vs. weakly connected to each other at the base and sometimes weakly branching), 20-35 lateral veins per side (vs. 35–65 per side), blades decurrent at the geniculum (vs. broadly cuneate to truncate at base) and a thin, distally undulate petiole sheath (vs. petiole sheath never undulate).

**Figure 2.**
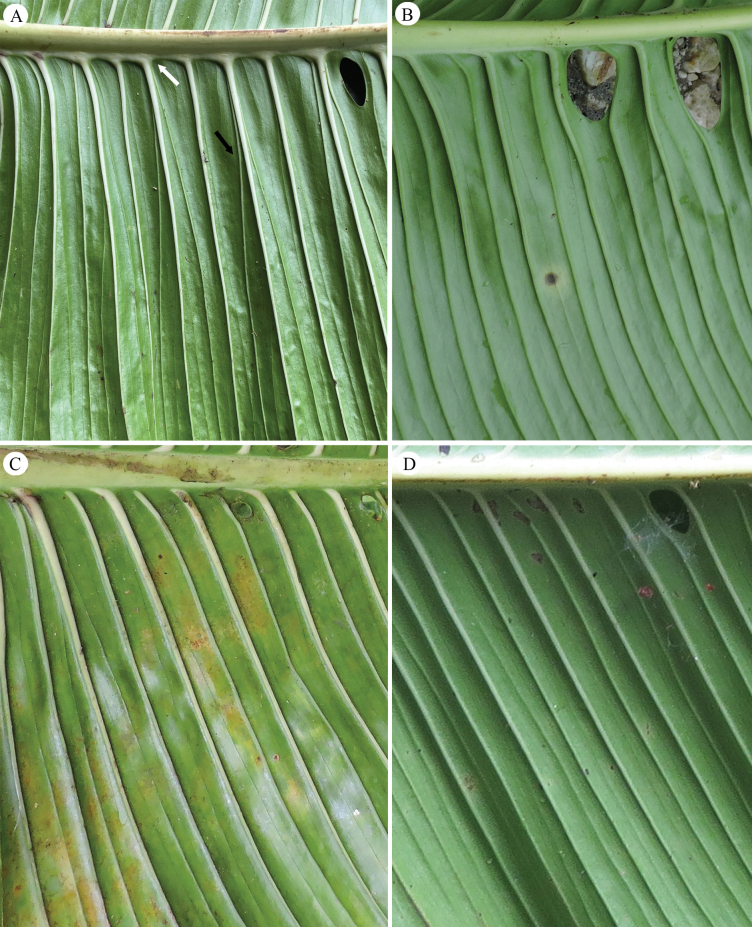
Patterns of primary lateral veins of *Monstera corana* M. Cedeño & O. Ortiz and similar species. A. *M. corana* with primary lateral connected to each other at the base (white arrow), thick and white up to half its length, with one or two branching veins that start from the base (black arrow); B. *M. buseyi* with primary lateral veins green and weakly branching; C. *M. alfaroi* with primary lateral veins white and not branched; D. *M. costaricensis* with primary lateral veins white and not branched. Photos by M. Cedeño.

##### Description.

Robust appressed-climbing nomadic vine. SEEDLINGS: unknown. JUVENILE PLANTS: unknown. ADULT PLANTS: root climbers; ***stem*** cylindrical, minutely white-green warty, looking like a pulverulent indument ; ***internodes*** 0.5–7 cm long, 4–7 cm diam.; ***anchor roots*** blackish; ***feeder roots*** blackish; ***petiole*** 50–75 cm long, green, minutely white punctulate especially towards the base, minutely white-green warty the rest of its length (like the stem), sheathed along its entire length; ***petiole sheath*** thin, persistent and undulate from the middle of the petiole to the geniculum, deciduous from middle to the base; ***geniculum*** minutely white-green warty throughout, flattened adaxially and transversally convex abaxially, 5.0–7.5 cm long; ***blades*** 50–80 × 40–55 cm, narrowly ovate, rounded or asymmetric at base, shortly acuminate at the apex, membranaceous, decurrent on the geniculum and almost connecting with petiole sheath; ***mid-rib*** white, concave adaxially, square abaxially; ***primary lateral veins*** 20–35 per side, impressed adaxially, prominent abaxially the base of each vein abruptly prominent, connected to each other at the union with the main vein, thick and white up to half their length, diverging at 80–90° and often with one or two subsidiary (branching) veins starting from the base or close to the base, on either side of the primary lateral vein, running near the primary vein and then separate until they reach the margin; ***secondary veins*** parallel to the primary lateral veins; ***collective vein*** poorly visible; ***fenestrations*** scarce, near the mid-rib, when present in two rows along the mid-rib, comprising small oblong holes 0.5–4 cm long, often scattered towards the margin; ***margins*** entire. INFLORESCENCES solitary, produced on ascending stems; ***peduncle*** 25 cm long, minutely white-green verrucose; ***spathe*** unknown; ***spadix*** 22 cm long, 5 cm diam.; ***basal sterile flowers*** 3–4 mm long; ***fertile flowers*** 5–7 mm long; stamens unknown; anthers unknown; ovary quadrangular in longitudinal section, 2–3 mm long, style truncate; stigma linear; ***berries*** with the stylar cap after anthesis white, white-cream when ripe; pulp white; ***seeds*** unknown.

**Figure 3.**
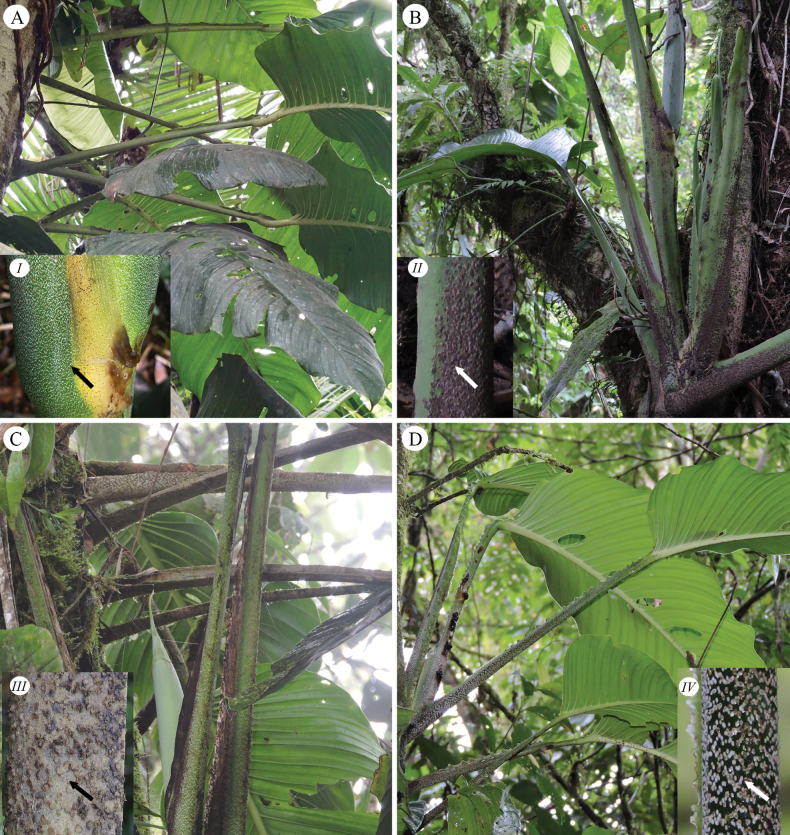
Petioles ornamentation of *Monstera corana* M. Cedeño & O. Ortiz and similar species. A. Adult plant of *Monstera corana* with petiole sheath deciduous in the lower half of the petiole and undulate in the upper half to near the base of the geniculum; (*I*) petiole green punctulate at the base with green and white punctae; B. Adult plant of *M. buseyi* with petiole sheath deciduous; (*II*) petiole green black verrucose; C. Adult plant of *M. alfaroi* with petiole sheath deciduous; (*III*) petiole brown with brown and black verrucose; D. Adult plant of *M. costaricensis* with petiole sheath undulate throughout its length; (*IV*) petiole with green and white verrucose. Photos by M. Cedeño.

##### Etymology.

The epithet “*corana*” refers to Cora Jane, the daughter of Dr. Sarah Spaulding, who has been a supporter of several projects about Aroid conservation in Costa Rica.

##### Distribution and ecology.

*Monstera corana* is endemic to Panama, known only from the type locality (Chiriquí Grande, Bocas del Toro Provinces) at 85 m elevation in a Tropical rain forest life zone.

##### Phenology.

Flowering unknown; mature fruits in November.

##### Discussion.

This species is a member of Monstera sect. Monstera and is characterised by its stem with densely and finely white-green warts, the green petiole white punctulate towards the base and densely and finely white-green warty towards the apex, the petiole sheath undulate from the middle of the petiole extending to the base of the geniculum, the blades narrowly ovate, rounded or asymmetric at base and weakly perforated near the mid-rib, primary lateral veins thick and white, connected to each other and the union with the main vein and abruptly prominent at the base, with subsidiary veins arising from the base or close to the base of the primary vein (Table 1).

**Table 1.** Overview of morphological characters of pachycaul *Monstera* species. Note: Leaf length = petiole length + leaf blade length; inflorescence length = peduncle length + spathe length.

Species	Leaf length (cm)	Leaf blade size (cm)	Inflorescence length (cm)	Diagnostic features	Distribution
*M. alfaroi*	100–160 cm	60–90 × 30–45	30–55	Unbranched primary lateral veins, fenestrations present, ovary quadrangular, style hexagonal, stigma linear.	Costa Rica
*M. colossica*	97–65 cm	47–70 × 28–40	35–54	Branched primary lateral veins, fenestrations absent or present, ovary rectangular, style hexagonal with a slightly conical stigmatophore, stigma linear.	Panama
*M. corana*	100–155	50–80 × 40–55	47	Branched primary lateral veins, fenestrations present, ovary quadrangular, style truncate, stigma linear.	Panama
*M. deliciosa*	40–222 cm	40–112 × 40–88	22–48	Unbranched primary lateral veins, fenestrations present, ovary quadrangular, style compressed and hexagonal with a cupuliform stigmatophore, stigma circular.	Southern Mexico, Guatemala
*M. gigas*	138–300	72–140 × 34–61	64–76	Unbranched primary lateral veins, blades entire, ovary rectangular, style hexagonal, stigma circular.	Panama
*M. lechleriana*	63–105 cm	47–75 × 31–49	22–52	Unbranched primary lateral veins, fenestrations present, ovary rectangular, style hexagonal, stigma circular.	South America
*M. titanum*	87–195	47–100 × 28–35	43–95	Unbranched primary lateral veins, fenestrations present, ovary rectangular, style pyramidal, stigma linear.	Panama

*Monstera corana* is most similar to *M. buseyi* which differs in having green petiole white punctulate toward the base and densely and finely white-green warts towards the apex (vs. petiole green with black warts), the petiole sheath distally undulate (vs. petiole sheath never undulate), leaf blade 50–80 × 40–55 cm, narrowly ovate, rounded or asymmetric at base (vs. leaf blade 27–75 × 11–33 cm, ovate to lanceolate-ovate or elliptic, broadly cuneate to truncate at base), and primary lateral veins 20–35 per side, white and strongly connected to each other at the base and always strongly branching (vs. primary lateral veins 35–65 per side, pale green and weakly connected to each other at the base and sometimes weakly branching) (Table 1).

*Monstera corana* is also similar to *M. alfaroi* and *M. costaricensis* (Engl. & K. Krause) Croat & Grayum. However, it differs from *M. alfaroi* in having the green petiole minutely white punctulate toward the base (vs. petiole brown at base, brown-black and white warty), the petiole sheath thin and distally undulate, persistent from half way to the geniculum or deciduous from half way towards the base (vs. petiole sheath undulate in the new leaf and later deciduous), primary lateral veins abruptly prominent at the base connected to each other, diverging at 80–90° and with two branching veins that start from the base or close to the base (vs. primary lateral veins never connected to each other at the base, diverging at 50–70° and never branching), leaf blade membranaceous, decurrent on the geniculum and almost connecting with petiole sheath, (vs. leaf blade coriaceous or subcoriaceous, not decurrent on the geniculum) and *Monstera corana* occurs at 85 m elevation in Tropical rain forest whereas *M. alfaroi* occurs at 1100–1250 m elevation in Lower montane rain forest life zone (Table 1).

*Monstera corana* differs from *M. costaricensis* in having the petiole sheath thin and distally undulate (vs. petiole sheath thick and undulate throughout its length), the leaf blade 40–55 cm wide, membranaceous and decurrent on the geniculum and almost connecting with petiole sheath (vs. leaf blade 15–35 cm wide, coriaceous or thinly coriaceous, slightly decurrent-undulate up to medial part of the geniculum), lateral veins abruptly prominent at the base connected to each other, diverging at 80–90° and with two branching veins that start from the base (vs. primary lateral veins never branching, departing at 45–65°) and the flower in the spadix with truncate style (vs. flower in the spadix with conic style) (Table 1). *Monstera corana* and *M. costaricensis* occur sympatrically in the tropical rain forest in Chiriquí Grande area.

#### ﻿ Monstera colossica

Taxon classificationPlantaeAlismatalesAraceae

M.Cedeño & O.Ortiz sp. nov.

D7003B35-1862-5711-A638-9899F17259DC

urn:lsid:ipni.org:names:77372987-1

Figs 4, 5

##### Type.

Panama • Provincia Chiriquí: distrito Gualaca, camino a Bosque Protector de Palo Seco, 1 September 2018, 1100 m elev., *O. Ortiz, H. Hentrich, M. Cedeño, P. Diaz-Jiménez, and R.M. Baldini 2730* (holotype PMA!).

**Figure 4.**
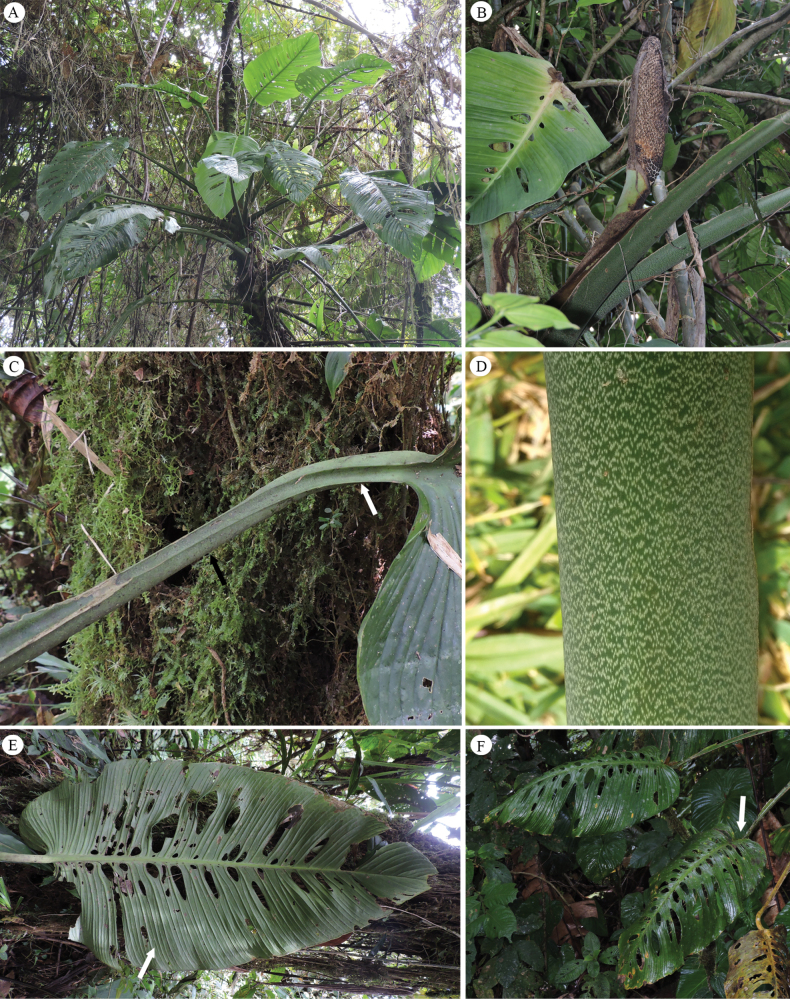
*Monstera colossica* M. Cedeño & O. Ortiz. A. Adult plant growing in the forest around Fortuna; B. Infructescence in development with remnants of the spathe; C. Free portion of the petiole sulcate adaxially (black arrow) and convex abaxially (white arrow); D. Petioles smooth and conspicuously light green punctulate; E. Lower surface of the adult leaf blade with many primary lateral veins (arrow) and two rows of sub-circular holes along the mid-rib; F. Adult leaf blade subcordate at base. Photos by M. Cedeño.

##### Diagnosis.

*Monstera colossica* differs from *M. titanum* in having primary lateral veins pale green abaxially and with 2–3 branches close to the mid vein (vs. primary lateral veins never branched and white), short peduncle 22 cm long (vs. 25–48 cm long), short spathe up to 32 cm long (vs. spathe up to 47 cm long), style hexagonal with stigmatophore slightly conical (vs. style pyramidal and distally cylindrical) and always appressed-climbing (vs. appressed terrestrial on rocks or appressed-climbing).

**Figure 5.**
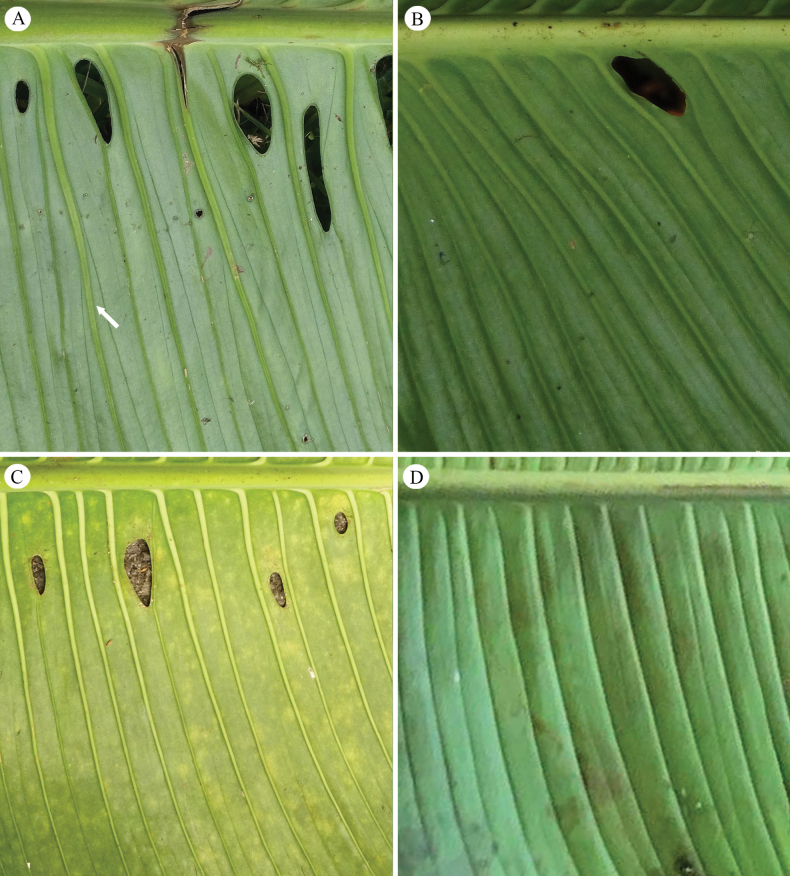
Patterns of primary lateral veins of *Monstera colossica* M. Cedeño & O. Ortiz and similar species. A. *Monstera colossica* with primary lateral veins branching with 2 or 3 branches; B. *Monstera oreophila* with primary lateral veins green and weakly branching; C. *Monstera titanum* with primary lateral veins white and not branched; D. *Monstera gigas* with primary lateral veins green and not branched. Photos by M. Cedeño.

##### Description.

Robust appressed-climbing nomadic vine. SEEDLINGS: unknown. JUVENILE PLANTS: root climbers; ***internodes*** 2.0–4.0 cm long, 5.0–7.0 mm diam.; petioles 22–30 cm long; sheathed up to ca. 5 cm before geniculum; ***leaves*** appressed to the phorophyte with visible petiole, ***blades*** 18–28 cm long, 12–18 cm wide, 1.3–1.5 times longer than broad, abruptly acuminate at apex; ***collective veins*** 3.0–5.0 mm from margin; ***margin*** entire or rarely pinnatilobed. ADULT PLANTS: root climbers; ***stems*** light or dark green, white punctulate, smooth, cylindrical; ***internodes*** 1.5–4.0 cm long, 4.0–7.0 cm diam., medium green, semi-glossy; ***petioles*** 50–65 cm long, densely and conspicuously white punctulate, especially towards base, equally as long as or 1.1 times longer than blades, sheathing to the base of the geniculum to ca. 10 cm below it, free portion sulcate; ***petiole sheath*** deciduous; ***geniculum*** sulcate adaxially and convex abaxially, 4.0–7.0 cm long; ***blades*** 47–70 × 28–40 cm, narrowly ovate-elliptic, mostly entire, more or less equilateral, subcoriaceous and slightly bicolorous, dark green and semi-glossy above, moderately paler and matte below, drying dark brown and weakly glossy above, greenish-yellow-brown and semi-glossy below, short-acuminate at apex, subcordate at base; mid-rib broadly sunken and concolorous above, narrowly rounded and paler below; ***primary lateral veins*** 20–25 per side, narrowly sunken above, convex and slightly paler green below, diverging at 50–70°, sometimes branching with two to three branches within 1.0–3.0 cm from the mid-rib; ***secondary veins*** parallel and reticulate towards the margin; ***collective vein*** visible; ***fenestrations*** absent or present; when present, in two or three rows along the mid-rib, usually comprising small sub-circular holes 1.0–5.0 cm diameter, near the mid-rib; ***margins*** entire, but sometimes the larger perforations tearing through to the margins. INFLORESCENCE solitary; ***peduncle*** 20–22 cm long; ***spathe*** 15–32 cm long, 25 cm wide when flattened, coriaceous, whitish; ***spadix*** 18–22 cm long, 3–4 cm diam., greenish; ***basal sterile flowers*** 4.0–5.0 mm long; ***fertile flowers*** 7.0–9.0 mm long; stamens 3–8 mm long, with laminar filaments; ovary rectangular in longitudinal section, 4.0–6.0 × 2.0–3.0 mm; style hexagonal and with stigmatophore slightly conical; stigma linear; ***berries*** with a creamy stylar cap during development, ripe berry colour unknown; ***seeds*** unknown.

##### Etymology.

The specific epithet “*colossica*” is derived from the Latin *colossus*, itself from the Greek *kolossos* (κολοσσός), meaning “giant statue” or “colossus”, highlighting the plant’s overall massive and striking appearance.

##### Distribution and ecology.

*Monstera colossica* is endemic to Panama, known only from the type locality on the border of Chiriquí and Bocas del Toro Provinces and in Cerro Colorado at 1450–1480 m elevation in a Premontane rainforest life zone.

##### Phenology.

Flowering time in November; mature fruits in June.

##### Discussion.

The species is a member of Monstera sect. Monstera and is characterised by its adult plants with an appressed-climbing habit, short internodes, minutely white punctulate and fully sheathed petioles with a sharply sulcate geniculum, large narrowly ovate-elliptic subcordate blades with two rows of small elliptic perforations on both sides and the primary lateral veins diverging at 50–70° and branching with 2–3 branches within 1.0–3.0 cm from the mid-rib.

*Monstera colossica* specimens were included as part of *M. titanum* in Cedeño-Fonseca et al. (2021) as paratypes, but this species differs from *M. titanum* in having primary lateral veins branching with 2–3 branches and pale green below (vs. primary lateral veins never branched and white), short peduncle 22 cm long (vs. 25–48 cm long), short spathe up to 32 cm long (vs. spathe up to 47 cm long), style hexagonal with stigmatophore slightly conical (vs. style pyramidal and distally cylindrical) and always appressed-climbing (vs. appressed terrestrial on rocks or appressed-climbing) (Table 1).

*Monstera colossica* could be confused with *M. oreophila* Madison, but it differs in having more robust stems, 5–7 cm in diameter, (vs. slender stems, 2–4 cm diam.), the petiole smooth and minutely white punctulate at the base (vs. petiole never punctulate, but warty or sometimes smooth), up to 25 primary lateral veins branching with 2 or 3 branches (vs. up to 45 primary lateral veins never branching), peduncle up to 22 cm long (vs. peduncle up to 40 cm long), spadix 18–22 × 3–4 cm diam. (vs. 7–15 × 1.3–3.0 cm) and the spathe whitish (vs. spathe pinkish externally and light orange-yellow internally) (Table 1).

*Monstera colossica* also could be confused with *M. gigas*, but differs in having short petioles 50–65 cm long (vs. 66–140 cm long), petiole sheaths extending to the geniculum (vs. petiole sheathed up to ca. 10–15 cm before geniculum, the free portion terete), smaller leaf blades 47–70 cm long (vs. leaf blades 72–140 cm long), up to 25 primary lateral veins branching with two–three branches from the mid-rib (vs. up to 65 primary lateral veins never branched) and the geniculum sulcate adaxially and convex abaxially (vs. geniculum completely terete) (Table 1). *M. colossica* and *M. gigas* grow sympatrically in the cloud forest in the Fortuna area.

##### Additional specimens examined.

Panama • Comarca Ngäbe-Buglé: Cerro Colorado, along mining road 31.6 km beyond bridge over Río San Felix (10.6 km beyond turnoff to Escopeta), 1690 m elev., 15 July, 1976, *T.B. Croat 37178* sub *Monstera titanum* (MO!); • 7 mi W of Chamé, along trail through Guaymí Village, 1500 m elev., 8 July 1988, *T.B. Croat 69190* sub *Monstera titanum* (MO!); • 9.2 mi W of Chamé, along trail E of road which leads down to stream, 1450–1480 m elev., 6 July 1988, *T.B. Croat 69033* sub *Monstera titanum* (MO!); • Cerro Colorado, 9.2 mi W of Chamé, along trail E of road which leads down to a stream, 1450–1480 m elev., 6 July, 1988, *T.B. Croat 69012* sub *Monstera titanum* (MO! PMA!); • Chiriquí: Distrito Gualaca, Reserva Forestal Fortuna, Divisora Continental, 1154 m elev., 9 November 2013, *O. Ortiz, J. Batista & F. Miranda 1809* sub *Monstera titanum* (PMA); • Chiriquí: Gualaca, Hornito, Fortuna, camino a Chiriquí Grande, 1230 m elev., *M. Cedeño et al. 2329* sub *Monstera titanum* (PAM!, USJ!); • Chiriquí: Gualaca, Hornito, Fortuna, camino a Chiriquí Grande, 780 m elev., 17 November 2022, *M. Cedeño et al. 2733* (PMA!, B!).

## Supplementary Material

XML Treatment for Monstera corana

XML Treatment for Monstera colossica

## ﻿Additional information

### Use of AI

No use of AI was reported.

### Author ORCIDs

Marco Cedeño-Fonseca https://orcid.org/0000-0003-0119-0427

Orlando O. Ortiz https://orcid.org/0000-0002-7805-0046

Alistair Hay https://orcid.org/0000-0003-2884-6430

Alejandro Zuluaga https://orcid.org/0000-0002-5874-6353

### Data availability

All of the data that support the findings of this study are available in the main text.
